# TCR repertoire sequencing identifies synovial Treg cell clonotypes in the bloodstream during active inflammation in human arthritis

**DOI:** 10.1136/annrheumdis-2015-208992

**Published:** 2016-06-16

**Authors:** Maura Rossetti, Roberto Spreafico, Alessandro Consolaro, Jing Yao Leong, Camillus Chua, Margherita Massa, Suzan Saidin, Silvia Magni-Manzoni, Thaschawee Arkachaisri, Carol A Wallace, Marco Gattorno, Alberto Martini, Daniel J Lovell, Salvatore Albani

**Affiliations:** 1 SingHealth Translational Immunology and Inflammation Centre, SingHealth and Duke-NUS Graduate Medical School, Singapore, Singapore; 2 Translational Research Unit, Sanford-Burnham Medical Research Institute, San Diego, California, USA; 3 Department of Pathology and Laboratory Medicine, University of California Los Angeles, Los Angeles, California, USA; 4 Department of Microbiology, Immunology and Molecular Genetics, University of California Los Angeles, Los Angeles, California, USA; 5 Second Pediatrics Division, University of Genoa and G Gaslini Institute, Genova, Italy; 6 Lab Biotecnologie, Fondazione IRCCS Policlinico San Matteo, Pavia, Italy; 7 Pediatric Rheumatology Unit, IRCCS Ospedale Pediatrico Bambino Gesù, Rome, Italy; 8 Duke-NUS Graduate Medical School and Rheumatology and Immunology Service, KK Women's and Children's Hospital, Singapore, Singapore; 9 Seattle Children's Hospital and Research Institute, Seattle, Washington, USA; 10 Division of Rheumatology, Cincinnati Children's Hospital Medical Center, and Department of Pediatrics, University of Cincinnati College of Medicine, Cincinnati, Ohio, USA

**Keywords:** Juvenile Idiopathic Arthritis, Rheumatoid Arthritis, Synovial fluid, T Cells

## Abstract

**Objectives:**

The imbalance between effector and regulatory T (Treg) cells is crucial in the pathogenesis of autoimmune arthritis. Immune responses are often investigated in the blood because of its accessibility, but circulating lymphocytes are not representative of those found in inflamed tissues. This disconnect hinders our understanding of the mechanisms underlying disease. Our goal was to identify Treg cells implicated in autoimmunity at the inflamed joints, and also readily detectable in the blood upon recirculation.

**Methods:**

We compared Treg cells of patients with juvenile idiopathic arthritis responding or not to therapy by using: (i) T cell receptor (TCR) sequencing, to identify clonotypes shared between blood and synovial fluid; (ii) FOXP3 Treg cell-specific demethylated region DNA methylation assays, to investigate their stability and (iii) flow cytometry and suppression assays to probe their tolerogenic functions.

**Results:**

We found a subset of synovial Treg cells that recirculated into the bloodstream of patients with juvenile idiopathic and adult rheumatoid arthritis. These inflammation-associated (ia)Treg cells, but not other blood Treg cells, expanded during active disease and proliferated in response to their cognate antigens. Despite the typical inflammatory-skewed balance of immune mechanisms in arthritis, iaTreg cells were stably committed to the regulatory lineage and fully suppressive. A fraction of iaTreg clonotypes were in common with pathogenic effector T cells.

**Conclusions:**

Using an innovative antigen-agnostic approach, we uncovered a population of *bona fide* synovial Treg cells readily accessible from the blood and selectively expanding during active disease, paving the way to non-invasive diagnostics and better understanding of the pathogenesis of autoimmunity.

## Introduction

T cell-mediated autoimmunity occurs when checkpoints of peripheral tolerance, including suppression by CD4^+^CD25^+^FOXP3^+^ regulatory T cells (Treg cells), fail to delete or otherwise inactivate self-reactive clonotypes.^1^ Treg cells can either develop in the thymus or differentiate from peripheral naive T cells stimulated under tolerogenic conditions;^2^ in either case, the expression of the transcription factor FOXP3 is essential for their development and suppressive function.^3–5^

Autoimmune rheumatic diseases, including both the adult form—rheumatoid arthritis (RA)—and the juvenile form—oligoarticular and polyarticular juvenile idiopathic arthritis (JIA)—are characterised by chronic joint inflammation and accumulation of autoreactive T cells in the synovium, with consequent tissue damage and joint degeneration.^6^ In recent years, it has become increasingly clear that failure of immune tolerance in rheumatic diseases is due to an imbalance between effector and regulatory mechanisms.^7^ ^8^

On the effector side, T cells from the inflamed joints are resistant to Treg cell-mediated suppression thanks to protein kinase B (PKB) hyperactivation in JIA.^9^ ^10^ In addition, we recently described a subset of circulating antigen-experienced, proinflammatory effector T (Teff) cells that are resistant to anti-tumor necrosis factor (TNF) therapy and are enriched in synovial clonotypes.^11^ Teff resistance to Treg cell-mediated suppression is also recognised in RA.^12^

As for the regulatory arm, Treg cells are believed to be reduced in numbers and dysfunctional in RA. Certain therapies, such as those relying on anti-TNF, can re-establish normal Treg cell counts and function.^13^ In JIA, discordant data have been reported on the prevalence of Treg cells in the blood, while most studies agree that they accumulate in the synovial fluid (SF).^14^ ^15^ In addition, synovial Treg cells seem able to suppress Teff proliferation and cytokine production in vitro, that is, once removed from the inflammatory *milieu*, suggesting that they are not intrinsically dysfunctional.^16^ However, no data are available on how current therapies affect Treg cell phenotype and function in JIA. In this regard, recent data demonstrate that Treg cells are a heterogeneous population. Indeed, Treg cells comprise both activated CD45RA^−^FOXP3^hi^ cells with enhanced suppressive function and turnover as well as quiescent CD45RA^+^ cells.^17^ Another Treg cell subset, defined by the expression of HLA-DR, is endowed with early contact-dependent suppressive function.^18^ Dissecting the role of the different subsets in disease pathogenesis is crucial to understand the immunological mechanisms underlying unresponsiveness to therapy and to develop more effective and targeted treatment regimens, which is the main goal of precision medicine. Unfortunately, data are often lacking in human disease due to limitations in the number and amount of available samples.

In this work, we demonstrate that a subset of *bona fide*, antigen-stimulated and suppressive Treg cells expands during active autoimmunity in JIA. Investigation of their TCR repertoire through next-generation sequencing demonstrates that these Treg cells are enriched in synovial clonotypes, some of which were shared with pathogenic Teff cells. Our work uncovers an easily accessible pool of synovial Treg cells in the circulation that might be used as a novel non-invasive diagnostic tool, and proposes an innovative antigen-agnostic approach to shed light on pathogenic mechanisms of autoimmune disease and unresponsiveness to therapy.

## Materials and methods

### TCR sequencing

TCRβ CDR3 sequencing was performed by Adaptive Biotechnologies. Statistical analysis of TCR repertoire datasets is described in detail in previous reports.^11^ Briefly, each sample is interpreted as a distribution of individuals (T cell genomes) belonging to different species (TCR clonotypes). The similarity between samples is determined by the number and frequencies of shared clonotypes, using either productive nucleotide sequences or amino acid sequences derived by *in silico* translation. The similarity between samples was calculated either using the Chao-modified Jaccard index, which varies from 0 (complete dissimilarity) to 1 (complete similarity), or by repeated random subsampling at equal sample size (ie, equal number of T cell genomes). The median percentage of clonotype overlap resulting from 200 subsamples was then plotted. Hierarchical clustering with single linkage and t-SNE dimensionality reduction of TCR repertoires were performed using the Chao-modified Jaccard index.^11^ ^19^ TCR repertoire diversities were determined using the Renyi index upon sample size normalisation across a range of values of the α parameter, which puts more weight on abundant (α>1) or rare (α<1) clonotypes.

Additional methodological details are available as online supplementary information.

10.1136/annrheumdis-2015-208992.supp1 Supplementary data

## Results

### A subset of Treg cells is more represented in patients with JIA unable to control inflammation

**Figure 1:**
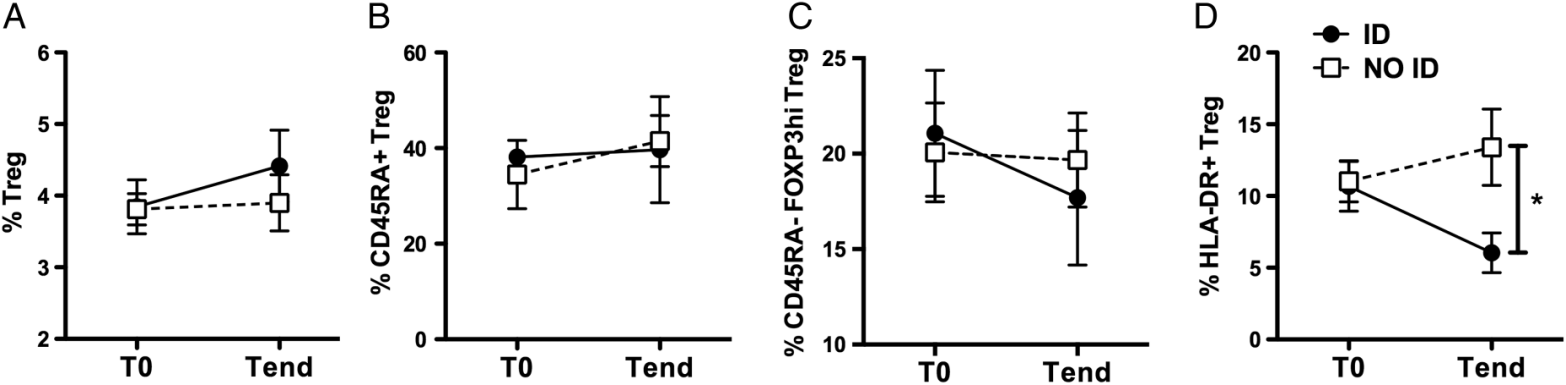
A subset of regulatory T (Treg) cells is more represented in patients with juvenile idiopathic arthritis (JIA) unable to control inflammation. (A–C) Frequency of total Treg cells in blood CD4^+^ T cells (A), and frequency of CD45RA^+^ (B), CD45RA^−^FOXP3^hi^ (C) or HLA-DR^+^ (D) in Treg cells of patients with JIA. All patients were NO ID at T0, and were segregated based on their clinical activity at Tend. ID: (prospective) inactive disease; NO ID: (prospective) active disease. Vertical lines represent SEM. n=10–13 per group, per time point. *p<0.05 (two-tailed unpaired t-test).

We investigated the phenotype of Treg cells in peripheral blood samples of patients with JIA, collected before (T0) and after (Tend) therapy,^20^ and stratified for responsiveness to therapy based on whether they reached inactive disease (ID)^21^ or not (NO ID) at Tend. All patients were NO ID at T0 but were classified as prospective ID or prospective NO ID based on their clinical activity at Tend. The percentage of Treg cells was similar between ID and NO ID patients, both before (ie, would be ID and would be NO ID, respectively) and after therapy (figure 1A).

We explored whether previously described subsets of Treg cells varied with clinical activity. The percentage of naive CD45RA^+^ Treg cells was identical between ID and NO ID patients, irrespective of the time point analysed (figure 1B). The prevalence of activated CD45RA^−^FOXP3^hi^ Treg cells was also similar between the two groups (figure 1C). By contrast, the percentage of HLA-DR^+^ Treg cells substantially decreased in ID while slightly increasing in NO ID patients over the course of the treatment, resulting in a more than doubled frequency of these inflammation-associated (ia)Treg cells in NO ID patients as compared with ID patients at Tend (figure 1D). Based on these data, we hypothesised that the size of the iaTreg cell subset is dynamically regulated: it expands during inflammation (ie, both before therapy and in patients failing therapy), likely in an effort to control autoreactivity, and it shrinks upon clinical improvement (ie, in patients who reach ID upon treatment). Therefore, iaTreg cells might be envisioned as a novel tool to track responsiveness to therapy.

### iaTreg cells are *bona fide* Treg cells endowed with suppressive ability

**Figure 2:**
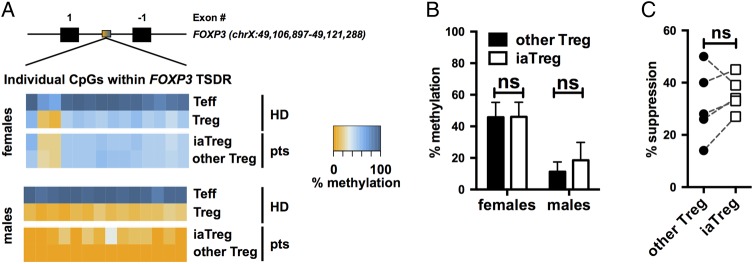
iaTreg cells are *bona fide* Treg cells endowed with suppressive ability. (A) Methylation percentages of individual CpG sites in the *FOXP3* the Treg cell-specific demethylated region (TSDR), colour-coded according to the legend, in Teff and Treg cells from representative male and female NO ID patients. As a reference, the methylation profiles of cells from representative male and female healthy donors (HD) out of six are reported. Treg cells are partially demethylated in females due to X-inactivation. Absolute coordinates of the *FOXP3* gene according to the GRCh37/hg19 human genome assembly are indicated. (B) Summary of methylation percentages of iaTreg and other Treg cells (n=4 females and 4 males). (C) Teff were activated with anti-CD3/CD28 and cocultured with either iaTreg or the rest of circulating Treg cells. Suppression of Teff activation relative to a no-Treg cell control is reported. Each line corresponds to a patient. ns: not significant (two-tailed paired t-test). Teff, effector T; Treg, regulatory T; iaTreg, inflammation-associated regulatory T.

To determine whether iaTreg cells are truly suppressive cells, rather than Teff transiently upregulating FOXP3, we investigated their commitment to the regulatory lineage by analysing the methylation profile of the Treg cell-specific demethylated region (TSDR) within the *FOXP3* locus.^22^ ^23^ Unlike FOXP3 expression, this epigenetic feature is absent in unstable Treg cells and in FOXP3^+^ Teff.^24^ Both iaTreg cells and the rest of Treg cells were as demethylated at their TSDR as Treg cells from healthy donors (HD, figure 2A, B), indicating that they are *bona fide* regulatory cells.

Previous reports indicate that HLA-DR expressing Treg cells from HD are endowed with early contact-dependent suppressive function.^18^ We found that also iaTreg cells from patients with JIA are suppressive, to the same extent as the rest of Treg cells (figure 2C).

Altogether, these findings support the notion that iaTreg cells are genuinely tolerogenic and functionally competent.

### iaTreg cells are activated Treg cells able to recirculate through inflamed sites

**Figure 3:**
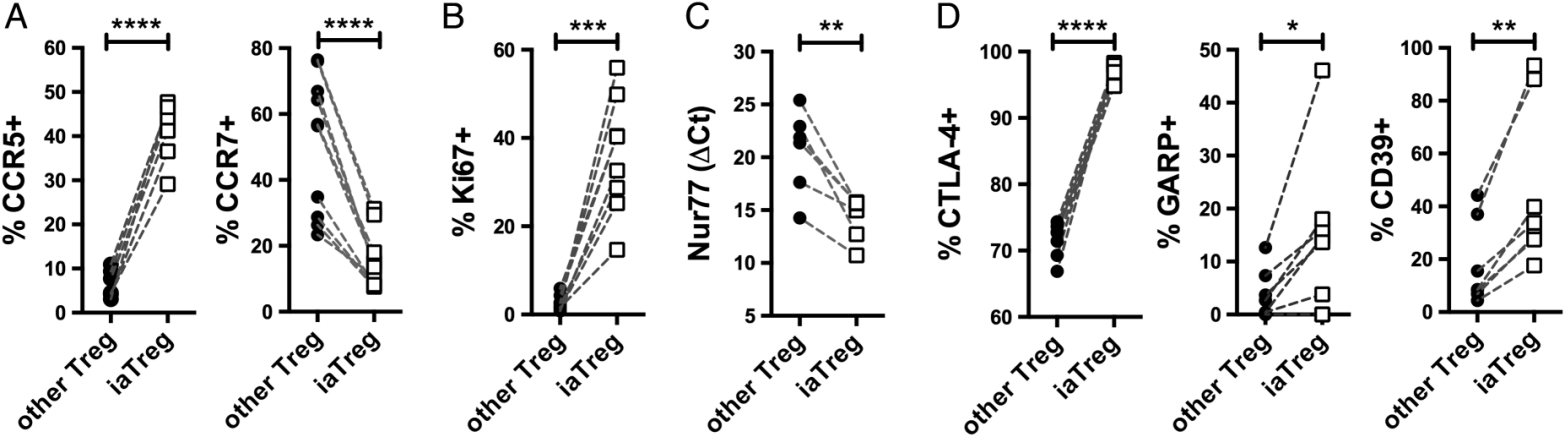
iaTreg cells are activated Treg cells able to recirculate through inflamed sites. Differential marker expression by flow cytometry (A, B and D) or qPCR (C) between iaTreg and other circulating Treg cells from NO ID patients at baseline. Each line corresponds to a patient. *p<0.05; **p<0.01; ***p<0.001; ****p<0.0001 (two-tailed paired t-test). Treg, regulatory T; iaTreg, inflammation-associated regulatory T.

Based on the observation that iaTreg cells expand during active autoimmunity and are fully suppressive in vitro, we hypothesised that these cells increase in numbers in an attempt to restrain deranged effector mechanisms. To address this hypothesis, we first characterised their migratory potential by looking at chemokine receptors. Compared with the other Treg cells, a larger fraction of iaTreg cells expressed the inflammatory tissue-homing receptor CCR5, as opposed to the lymph node-homing receptor CCR7 (figure 3A). This pattern of expression of chemokine receptors suggests that iaTreg cells have recently encountered the antigen, either in the lymph node upon priming, or in the synovium upon reactivation. In either case, antigen recognition would induce iaTreg cell activation and proliferation; therefore, we investigated Ki67 (which marks active cell cycle), Nur77 (an early marker of TCR signalling)^25^ and receptors endowed with regulatory function, including cytotoxic T-lymphocyte-associated protein-4 (CTLA-4), glycoprotein A repetitions predominant (GARP) and CD39.^1^ Consistent with our hypothesis of recent antigen encounter, a large proportion of iaTreg cells expressed Ki67 (figure 3B). iaTreg cells also upregulated Nur77, indicating that iaTreg cell proliferation is not due to bystander activation but to TCR triggering (figure 3C). In addition, when compared with the rest of Treg cells, many more iaTreg cells displayed an activated phenotype, as indicated by the induction of regulatory markers (figure 3D).

Altogether, our data suggest that iaTreg cells have recently proliferated and become activated upon cognate antigen encounter, and that they can recirculate between the blood and the inflamed peripheral tissues.

### iaTreg cells are enriched in synovial Treg cell clonotypes

To obtain conclusive evidence that blood iaTreg cells are indeed migrating to (or escaping from) the synovium, we investigated their TCR repertoires through next-generation sequencing of CDR3 regions within the TCRβ chains (see online supplementary table S1). This approach enabled us to determine at single-cell level whether iaTreg cells shared a substantial fraction of clonotypes with synovial Treg cells.

**Figure 4:**
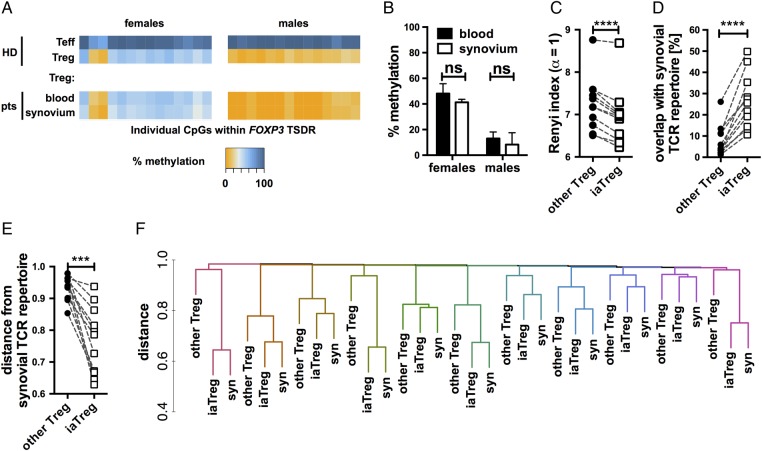
iaTreg cells are enriched in synovial Treg cell clonotypes at amino acid level. (A) Methylation percentages of individual CpG sites in the T cell-specific demethylated region (TSDR), colour-coded according to the legend, in Teff and Treg cells from representative male and female NO ID patients. (B) Summary of methylation percentages of blood and synovial Treg cells (n=7 females and 4 males). (C–F) Next-generation sequencing of TCRβ CDR3 sequences was performed on blood or synovial Treg cells of patients who had juvenile idiopathic arthritis (JIA) with active disease. All panels were built from *in silico*-translated (amino acid) sequences. (C) Summary of Renyi diversity indices of the TCR repertoires of iaTreg cells and the rest of blood Treg cells at α=1. (D) Overlap of the TCR repertoires of iaTreg cells and the rest of blood Treg cells with that of synovial Treg cells at equal sample size. (E) Summary of the pairwise distances between the TCR repertoires of iaTreg cells (or the rest of blood Treg cells) and those of synovial Treg cells, computed as 1-Chao-modified Jaccard index. (F) Unsupervised hierarchical clustering based on TCR repertoire distances. Each colour represents an individual patient. ***p<0.001; ****p<0.0001 (two-tailed paired t-test). HD, healthy donors; Teff, effector T; Treg, regulatory T; iaTreg, inflammation-associated regulatory T.

We first confirmed that synovial Treg cells are committed to the regulatory lineage by probing the epigenetic state of their TSDR. Synovial Treg cells were as demethylated as blood Treg cells, and comparable to Treg cells from HD (figure 4A, B), demonstrating that they were *bona fide* regulatory cells.

10.1136/annrheumdis-2015-208992.supp2 Supplementary table

We then investigated the diversity of the TCR repertoire of iaTreg cells using *in silico*-translated (amino acid) and nucleotide sequences, both yielding similar results (see figure 4C and online supplementary figure S2). iaTreg cells displayed reduced TCR diversity (lower Renyi index) compared with other Treg cells, in line with the notion that iaTreg cells have expanded in response to antigen stimulation. Interestingly, in some patients iaTreg cells were as oligoclonal as synovial Treg cells (see online supplementary figure S2).

10.1136/annrheumdis-2015-208992.supp3 Supplementary figures

Finally, we investigated the TCR repertoire similarity between synovial and iaTreg cells (figure 4D, F and online supplementary figure S3) using two different strategies.^11^ First, we determined the repertoire overlap between matched blood and synovial samples. Synovial Treg cells shared a substantially higher fraction of CDR3 sequences with iaTreg cells than with the rest of circulating Treg cells (see figure 4D and online supplementary figure S3A), demonstrating that iaTreg cells are enriched in synovial clonotypes. Second, we investigated the overall repertoire similarity across the three cell types by using the Chao-modified Jaccard index, which takes into account both the number of shared species and their frequencies. This approach supported our previous conclusion that the clonotypes of iaTreg cells are more closely related to synovial Treg cells than to the rest of circulating Treg cells (see figure 4E and online supplementary figure S3B). Consistently, unsupervised clustering based on this index segregated iaTreg cells apart from the rest of blood Treg cells but together with synovial Treg cells within each patient (see figure 4F and online supplementary figure S3C).

In summary, two independent analytical strategies led to the conclusion that iaTreg cells comprise clonotypes recirculating between the blood and the synovium.

### The TCR repertoire of iaTreg cells is donor-specific and partially shared with that of pathogenic Teff

In an effort to understand intraindividual and interindividual variability of TCR rearrangements, we asked whether the TCR repertoire of iaTreg cells shows convergence across patients, and whether it is closer to the effector (suggestive of transdifferentiation) or the regulatory lineage. To this aim, we compared the TCR repertoires of blood and synovial Teff and Treg cells. Blood Treg cells were segregated in iaTreg and other Treg cells. Blood Teff was similarly segregated in circulating pathogenic-like lymphocytes (CPLs) or other Teff.^11^ We used a dimensionality reduction algorithm (t-Distributed Stochastic Neighbor Embedding (t-SNE)) to produce a broad visualisation of the similarities across TCR repertoires (see online supplementary figure S4A). TCR repertoires clustered primarily by patient, confirming that TCR repertoires vary widely across individuals, as also indicated by hierarchical clustering (figure 4F). Then, within each patient, T cells clustered by lineage (Teff vs Treg cells).

Given the large differences across patients, we then visualised similarities across cell subsets within each subject by plotting all the pairwise distances between TCR repertoires as a heatmap. By far, the greatest similarity was observed between iaTreg cells and synovial Treg cells, and between CPLs and synovial Teff, respectively (see online supplementary figure S4B). Interestingly, we observed a gradient of TCR repertoire similarities between iaTreg cells and the other T cell subsets (see online supplementary figure S4C). Indeed, iaTreg cells were most closely related to synovial and other blood Treg cells, but distinct from non-CPL blood Teff cells. In between these two extremes, iaTreg cells displayed some degree of overlap with arthritis-associated synovial Teff and blood CPLs.

Altogether, our data suggest that the TCR repertoire of iaTreg cells is largely private across patients and ontogenically related to the Treg cell lineage. However, iaTreg cells share a detectable fraction of TCRs with Teff subsets connected to arthritis, whereas the bulk of ‘arthritis-inert’ blood Teff and Treg cells show negligible TCR repertoire overlap with each other (see online supplementary figure S4B).

### iaTreg cells are increased in the blood of patients with active RA

**Figure 5:**
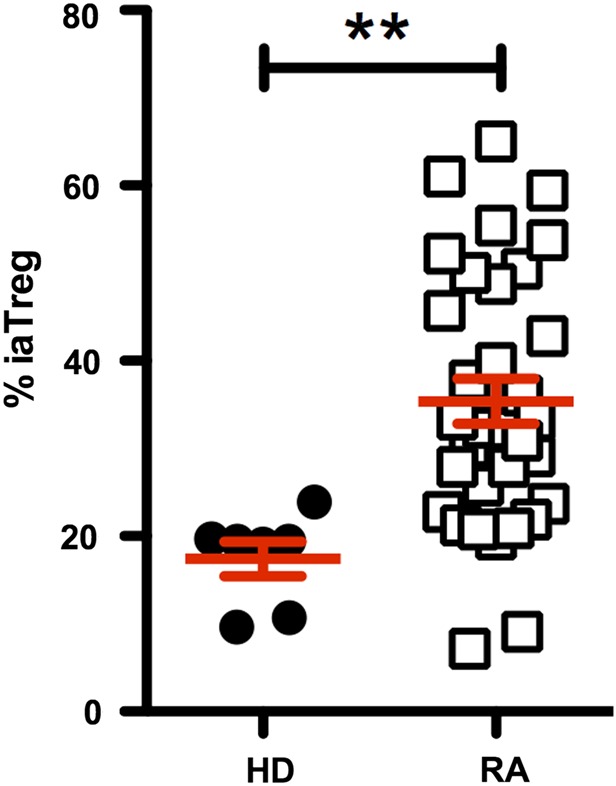
iaTreg cells are increased in the blood of patients with active rheumatoid arthritis (RA). Frequency of iaTreg cell within the whole Treg cell population in the blood of adult patients who had active RA (DAS28-3>3.2). Each dot corresponds to a sample. Horizontal red lines represent means, and error bars represent SEM. n=7 healthy donors (HD) and 33 patients with RA. **p<0.01 (two-tailed unpaired t-test). Treg, regulatory T; iaTreg, inflammation-associated regulatory T.

To address the question of whether iaTreg cells are associated with active inflammation in JIA, and also in other pathological conditions where effector mechanisms prevail over tolerance, we tested their frequency in the blood of adult patients who had active RA. When compared with healthy controls, patients with RA displayed an impressive expansion of circulating iaTreg cells (figure 5). Therefore, iaTreg cells are overrepresented during active inflammation in both adult and juvenile autoimmune arthritis.

## Discussion

In this work, we found that a subset of Treg cells is more prevalent in the blood of patients with JIA and active inflammation compared with patients achieving ID. This subset comprises *bona fide* activated and suppressive Treg cells able to recirculate between the systemic circulation and the synovial microenvironment. Our work provides several conceptual advances empowering future investigations in the field of translational research at large.

First, while Treg cells as a whole, as well as the naive (CD45RA^+^) and the activated CD45RA^−^FOXP3^hi^ Treg cell subsets, are stable over the course of treatment, iaTreg cells selectively persist in NO ID patients. Thus, abnormal features arising in disease may be overlooked when focusing on unsegregated populations, as the signal from (potentially small) relevant subsets would be diluted by a large pool of clinically irrelevant cells. This issue is critical, as most studies perform bulk measurements (eg, transcriptomic profiling) on whole blood Treg cells. In light of our data, we express concern that these efforts may be unfruitful until the cell target is redefined.

Second, mouse T cells do not express MHC class II.^26^ This lack severely limits the ability to experiment with iaTreg cells in vivo, and is likely a reason why their role in autoimmune arthritis has been overlooked so far. This disconnect underscores that both animal and human research are necessary to understand mechanisms of disease.

Third, the approach we devised to demonstrate the relevance of iaTreg cells in autoimmunity is a conceptual shift from traditional methods, which rely on antigen-based screening. These approaches, originally developed for conventional T cells and hindered by the low frequency of lymphocytes specific for any given antigen, are even more challenging with Treg cells, due to their low frequency in blood and the paucity of known Treg cell-recognised self-antigens. These hurdles, coupled with the small amount of blood withdrawn from juvenile patients, make these approaches ill-suited to investigate Treg cell reactivity. Rather than relying on prior antigen knowledge, we moved from the observation that arthritogenic clonotypes are enriched in the synovium.^27–34^ Thus, we compared synovial and circulating TCR repertoires at clonal resolution using next-generation sequencing. We demonstrated that iaTreg cells are enriched in clonotypes typical of synovial Treg cells, to the point that they cosegregate with synovial Treg cells rather than with the other blood Treg cells in unsupervised clustering. Our observations endow HLA-DR with the novel role of pinpointing a reservoir of synovial Treg cells in the peripheral circulation. Importantly, TCR repertoires were largely private across individuals, consistently with the mechanisms underlying HLA-driven positive selection of newly rearranged TCRs. As such, the identification of arthritis-associated clonotypes will likely require *de novo* investigations of immune features distinctive of each subject. iaTreg cells can empower such personalised investigations from simple blood draws rather than expensive and increasingly rare SF samples.

Lastly, iaTreg cells are expanded in active JIA, and also in adult RA. The consistency of our findings in two distinct autoimmune diseases highlights the clinical relevance of iaTreg cells in conditions where immune regulation is ineffective. Further investigations of this subset may yield critical insights into the pathological mechanisms at play in human arthritis and, possibly, autoimmunity at large.

We have recently reported that a subset of recently activated, proinflammatory Teff (CPLs) is expanded in this very same cohort of patients resistant to anti-TNF therapy.^11^ As effector and regulatory mechanisms are unbalanced during active arthritis, we initially expected that the observed increase in activated Teff would be matched by a corresponding decrease in Treg cells. However, Treg cells as a whole do not differ between ID and NO ID patients, but iaTreg cells are even expanded during active inflammation. In addition, iaTreg cells and CPLs display a similar expression pattern of chemokine receptors and markers of activation. Although these findings are conceivable, as these surface molecules are involved in pathways of T cell activation, differentiation and migration common to Teff and Treg cells, they raise the question of whether iaTreg cells are genuine regulatory cells. Our data show that iaTreg cells (i) are fully committed to the regulatory lineage, as demonstrated by their TSDR demethylation, (ii) express high levels of membrane-bound proteins associated with contact-dependent regulatory functions, such as CTLA-4,^35^ (iii) are functionally suppressive and (iv) are ontogenically related to the Treg cell lineage. Our results are consistent with previous data from normal donors demonstrating that HLA-DR^+^ Treg cells are endowed with early contact-mediated suppressive ability.^18^ Therefore, iaTreg cells do not appear intrinsically dysfunctional or epigenetically unstable.

Interestingly, iaTreg cells are actively proliferating upon TCR triggering in the absence of overt infection, as indicated by high prevalence of Ki67, reduced TCR repertoire diversity and Nur77 upregulation. In addition, although iaTreg cells are ontogenically related to Treg cells, they show some degree of TCR repertoire overlap with arthritis-associated synovial Teff and blood CPLs. This finding fits into the accepted paradigm whereby modestly self-reactive TCRs escaping negative selection give rise to either tolerogenic Treg cells or effector clonotypes poised for autoimmunity.^36^ The expansion of iaTreg cells during active inflammation may thus represent an attempt to counteract the growth of pathogenic Teff. However, the clinical outcome of NO ID patients indicates that iaTreg cells are unsuccessful, possibly because of extrinsic factors known to contribute to the inability of Treg cells to suppress in vivo, such as the proinflammatory cytokine environment in the inflamed synovium^15^ and the Teff resistance to Treg cell-mediated suppression.^8–10^ ^37^ Therefore, normalising the inflamed synovial microenvironment and the hyperactivation of the effector arm might help re-enable deranged tolerogenic mechanisms.
