# Supplementary material for: TCR repertoire sequencing identifies synovial Treg cell clonotypes in the bloodstream during active inflammation in human arthritis
Source: Ann Rheum Dis. 2016 Jun 16;76(2):435–41. doi: 10.1136/annrheumdis-2015-208992 (PMC5284348; doi:10.1136/annrheumdis-2015-208992)
Supplement: Supplementary data [file annrheumdis-2015-208992supp.pdf]

SUPPLEMENTARY METHODS

Samples

EDTA-anticoagulated blood from 30 poly-articular JIA patients before (T0) and after (Tend) at least 6 months of treatment with methotrexate, etanercept and prednisolone (tapered to zero in 4 months) was collected from participants in the TREAT study[1]. Patients were evaluated for clinical activity as described[1, 2]. Briefly, patients achieved inactive disease (ID) if they had: no joints with active arthritis; no fever, rash, serositis, splenomegaly, or generalized lymphadenopathy attributed to JIA; no active uveitis; normal ESR/CRP level; physician's global assessment of disease activity score as low as possible (score of 0)[2]. Patients were included in the TREAT study only if they were naive to etanercept and did not fulfill the ID criteria at baseline. In our cohort, 14 patients reached ID at Tend, while 16 did not and are referred to as NO ID.

Matched blood and synovial fluid (SF) from 11 NO ID patients (3 with poly-articular, 8 with oligo-articular JIA) for TCR repertoire investigation were collected at the G. Gaslini Institute, Genoa, and IRCCS Policlinico S. Matteo Foundation, Pavia (Italy). The median age and disease duration were 11.6 years (range: 2.4-22.9) and 7 years (range: 0.2-19.1), respectively. Of the 3 poly-articular patients, 2 were naive to therapy, while 1 was under methotrexate+infliximab therapy. Of the 8 oligo-articular patients, 6 were under methotrexate treatment, and 1 was naive to therapy. No information is available for the last patient.

Blood from 33 untreated RA patients with DAS28 in the moderate-to-high range (>3.2) was collected as part of the DNAJP1 study[3].

Blood from 7 healthy donors (HD) was collected at the TSRI Normal Blood Donor Service (La Jolla, CA).

Peripheral blood mononuclear cells (PBMCs) were separated by density gradient with Histopaque-1077 (Sigma-Aldrich) and frozen in freezing medium (90% FCS, 10% DMSO).

Flow cytometry

Upon thawing, cells were immediately stained. The Live/Dead Fixable Near-IR Stain (Life Technologies) was used to exclude dead cells. The FcR blocking reagent (Miltenyi Biotec) was used to block Fc receptors (FcR) before staining. The FOXP3 buffer set (eBioscience) was used to perform intracellular staining. The gating strategy for immunophenotyping is shown in **online supplementary Figure S1**. Antibodies were from Biolegend, BD Biosciences and eBioscience. FCS v3 files were analyzed with FlowJo (Treestar).

qPCR

RNA was isolated using the Picopure kit and quantified using the Quant-iT RiboGreen kit (both from Life Technologies). cDNA were then converted and amplified using the SMART-Seq v4 Ultra Low Input RNA Kit according to the manufacturer's instructions (Clontech). cDNA quality was checked on an Agilent Bioanalyzer 2100. qPCR was perfomed with SYBR green chemistry on a LightCycler 480 II (Roche). ΔCt values were calculated against a GAPDH reference. Primers were synthesized from Sigma-Aldrich in standard desalted format. Nur77 primer sequences were: FWD [GCACAGAAGAACTTCGGGAG]; REV [TCCCATATTGGGCTTGGATA].

TSDR methylation

gDNA was isolated with the ZR-Duet DNA/RNA MiniPrep kit, and bisulphite conversion was performed with EZ DNA Methylation-Direct kit (both from Zymo Research). FOXP3 TSDR methylation was assessed using a protocol optimized for scarce clinical samples, as described[4].

Suppression assay

In pilot experiments, we found significantly different viability between HLA-DR+ and HLA-DR- T cells after a traditional 5-day proliferation assay in vitro. To avoid the confounding effects of differential viability, we used the short 7-hr suppression protocol previously published[5]. Briefly, PBMCs were thawed and allowed to rest overnight in complete medium with 20 IU/ml rhIL-2. CD4+ T cells were sorted in CD14-CD4+CD25low/- Teff and HLA-DR-positive or -negative CD14-CD4+CD25highCD127- Treg cells. To discriminate Teff and Treg cells at the end

of the co-culture, Teff were labeled with low-dose (0.02 μM) CFDA-SE (Life Technologies). 5,000 Teff and 5,000 Treg cells were then stimulated with anti-CD3/CD28-coated beads (Teff:bead=5:1) in the presence of fluorochrome-conjugated anti-CD154. After 7 hrs of incubation, cells were stained with anti-CD69, Sytox Red (Life Technologies) was added and cells were acquired with a FACSAria III (BD Biosciences). All conditions were performed in duplicate. Suppression was computed from the loss of activated (CD69+ and/or CD154+) Teff.

Statistical analyses

Paired t-tests were used in comparisons of matched blood vs synovial Treg cells, whereas unequal variance unpaired t-tests were used for ID vs NO ID and HD vs RA comparisons. Sidak adjustment was used for multiple comparison correction.

References

1. Wallace CA, Giannini EH, Spalding SJ, Hashkes PJ, O'Neil KM, Zeft AS, et al. Trial of early aggressive therapy in polyarticular juvenile idiopathic arthritis. Arthritis Rheum. 2012 Jun; 64(6): 2012-2021.
2. Wallace CA, Ruperto N, Giannini E. Preliminary criteria for clinical remission for select categories of juvenile idiopathic arthritis. J Rheumatol. 2004 Nov; 31(11):2290-2294.
3. Koffeman EC, Genovese M, Amox D, Keogh E, Santana E, Matteson EL, et al. Epitope-specific immunotherapy of rheumatoid arthritis: clinical responsiveness occurs with immune deviation and relies on the expression of a cluster of molecules associated with T cell tolerance in a double-blind, placebo-controlled, pilot phase II trial. Arthritis Rheum. 2009 Nov; 60(11):3207-3216.
4. Spreafico R, Rossetti M, van den Broek T, Jansen NJ, Zhang H, Moshref M, et al. A sensitive protocol for FOXP3 epigenetic analysis in scarce human samples. Eur J Immunol. 2014 Oct; 44(10): 3141-3143.
5. Canavan JB, Afzali B, Scotta C, Fazekasova H, Edozie FC, Macdonald TT, et al. A rapid diagnostic test for human regulatory T-cell function to enable regulatory T-cell therapy. Blood. 2012 Feb 23; 119(8):e57-66.
