# Supplementary material for: TCR repertoire sequencing identifies synovial Treg cell clonotypes in the bloodstream during active inflammation in human arthritis
Source: Ann Rheum Dis. 2016 Jun 16;76(2):435–41. doi: 10.1136/annrheumdis-2015-208992 (PMC5284348; doi:10.1136/annrheumdis-2015-208992)
Supplement: Supplementary table [file annrheumdis-2015-208992supp_table.pdf]

Supplementary Table 1. TCR sequencing statistics.

Patient ID	Cell type	# reads	# TCRb sequences	# Unique TCRb sequences	In-frame rearrangements (%)
1	iaTreg	88,857	1,720	1,485	76.8
	other Treg	874,900	35,695	32,525	79.2
	synovial Treg	174,773	1,855	1,517	76.0
2	iaTreg	227,300	2,885	2,293	77.7
	other Treg	855,283	20,445	16,969	79.3
	synovial Treg	337,811	3,382	1,510	74.4
3	iaTreg	140,893	1,721	1,532	77.7
	other Treg	1,079,811	27,610	25,259	80.4
	synovial Treg	225,407	4,373	2,593	79.3
4	iaTreg	367,307	2,866	2,061	72.6
	other Treg	1,293,168	47,610	41,482	79.3
	synovial Treg	675,044	5,840	4,126	73.7
5	iaTreg	1,299,181	28,380	25,294	80.7
	other Treg	1,346,506	78,455	69,677	79.0
	synovial Treg	981,697	8,685	2,789	70.8
6	iaTreg	67,075	2,149	1,676	74.0
	other Treg	1,146,279	62,618	57,340	80.3
	synovial Treg	323,531	8,456	4,253	75.1
7	iaTreg	44,680	982	802	73.6
	other Treg	788,966	15,698	12,858	79.2
	synovial Treg	546,441	13,110	3,564	77.7
8	iaTreg	76,064	2,188	1,704	79.3
	other Treg	574,179	10,860	10,274	83.6
	synovial Treg	630,479	14,147	4,530	81.1
9	iaTreg	45,926	1,291	991	71.4
	other Treg	603,784	20,384	15,918	79.4
	synovial Treg	355,230	10,650	4,964	76.7
10	iaTreg	56,126	930	840	72.7
	other Treg	946,801	21,672	20,085	77.8
	synovial Treg	387,119	8,014	3,700	75.0
11	iaTreg	58,911	1,533	1,169	74.2
	other Treg	984,143	29,782	22,295	79.8
	synovial Treg	415,301	11,796	4,820	78.7
