# Supplementary material for: TCR repertoire sequencing identifies synovial Treg cell clonotypes in the bloodstream during active inflammation in human arthritis
Source: Ann Rheum Dis. 2016 Jun 16;76(2):435–41. doi: 10.1136/annrheumdis-2015-208992 (PMC5284348; doi:10.1136/annrheumdis-2015-208992)
Supplement: Supplementary figures [file annrheumdis-2015-208992supp_figures.pdf]

SUPPLEMENTARY FIGURES

Supplementary Figure 1. Gating strategy for iaTreg cell immunophenotyping. Total PBMCs were thawed and immediately stained with a viability dye, CD14, CD4, CD3, CD25, FOXP3 and HLA-DR, and gated as shown. CD14 was included in the staining mix to exclude monocytes, which express CD4 at low levels.

Supplementary Figure 2. iaTreg cells display a less diverse TCR repertoire compared to other circulating Treg cells. Renyi diversity indices of the TCR repertoires of iaTreg cell and the rest of blood Treg cells for amino acid sequences over a range of α values.

Supplementary Figure 3. iaTreg cells are enriched in synovial Treg cell clonotypes at nucleotide level. Next-generation sequencing of TCRβ CDR3 sequences was performed on blood or synovial Treg cells of JIA patients with active disease. All panels were built from nucleotide sequences. **A.** Overlap of the TCR repertoires of iaTreg cell and the rest of blood Treg cells with that of synovial Treg cells at equal sample size. **B.** Summary of the pairwise distances between the TCR repertoires of iaTreg cell (or the rest of blood Treg cells) and those of synovial Treg cells, computed as 1 - Chao-modified Jaccard index. **C.** Clustering based on TCR repertoire distances. Each color represents an individual patient. *** when $p<0.001$; **** when $p<0.0001$ (two-tailed paired t-test).

Supplementary Figure 4. The TCR repertoire of iaTreg cells is donor-specific and partially shared with that of pathogenic Teff. Next-generation sequencing of TCRβ CDR3 sequences was performed on blood or synovial Teff and Treg cells of JIA patients with active disease. All panels were built from *in silico*-translated (amino acid) sequences. Pairwise TCR repertoire distances (computed as 1 - Chao-modified Jaccard index) between different subsets of Treg and Teff cells from 11 patients were either fed to a dimensionality reduction algorithm (t-SNE) (**A**) or plotted as heatmaps (**B**). In (**B**), both results from individual patients (bottom) and averages (top) are shown. (**C**) TCR repertoire distances between iaTreg

cells and Teff or Treg cell subsets. Each line represents a patient. * when $p<0.05$; ** when $p<0.01$; *** when $p<0.001$ (Sidak adjustment for multiple comparisons).

Figure S1

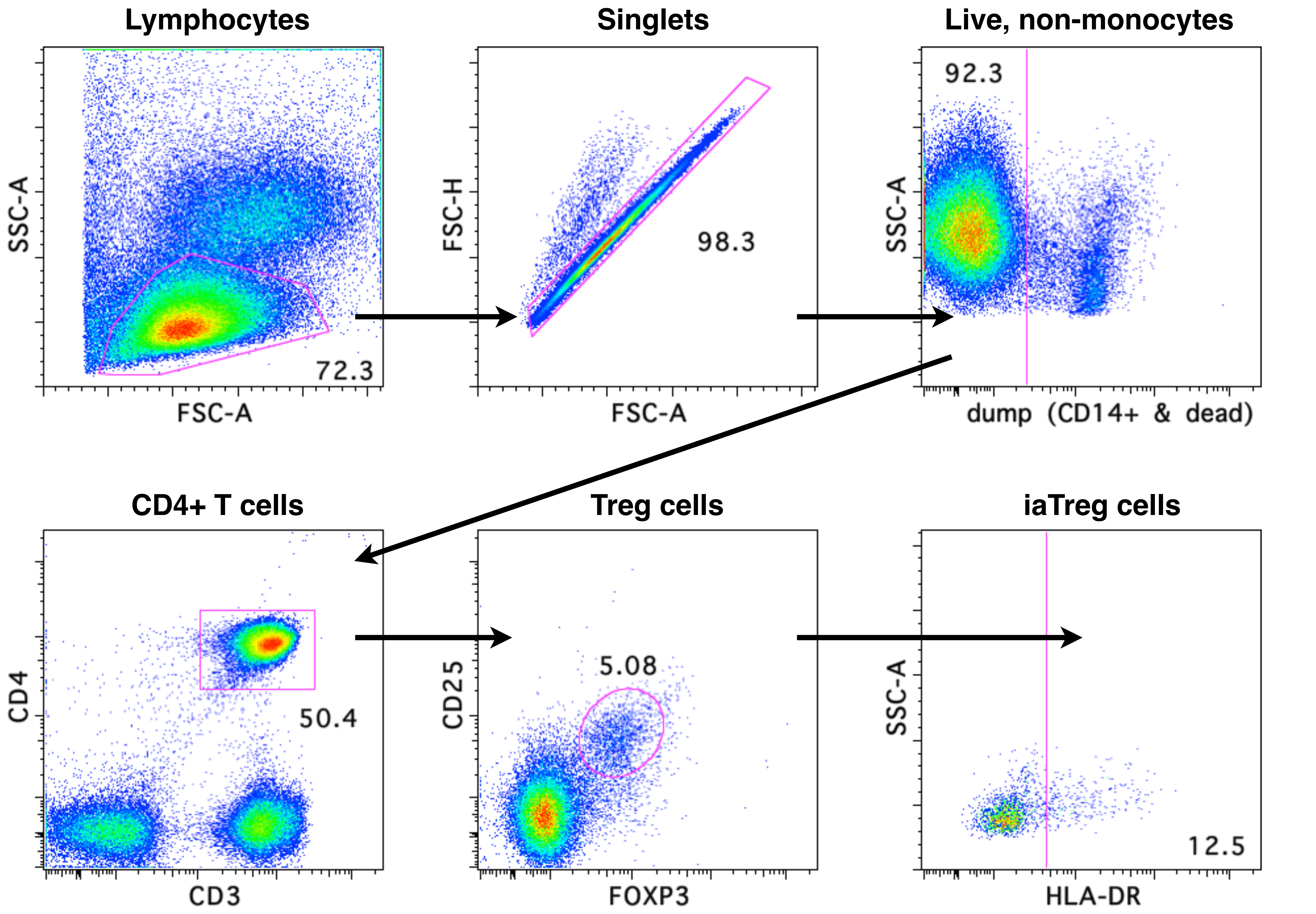
Lymphocytes
SSC-A
FSC-A
72.3
Singlets
FSC-H
FSC-A
98.3
Live, non-monocytes
92.3
SSC-A
dump (CD14+ & dead)
CD4+ T cells
CD4
CD3
50.4
Treg cells
CD25
FOXP3
5.08
iaTreg cells
SSC-A
HLA-DR
12.5

Figure S2

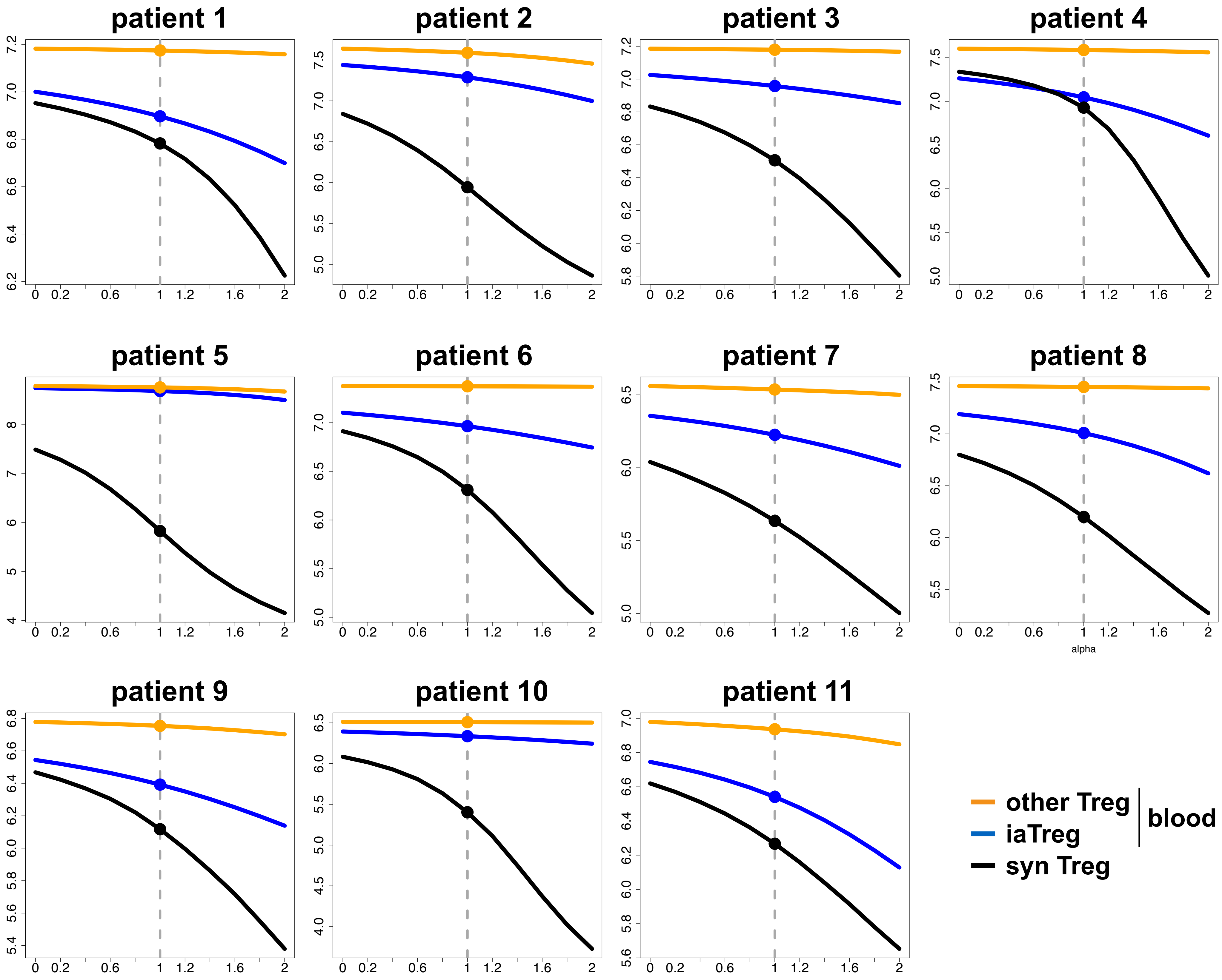
patient 1
patient 2
patient 3
patient 4
patient 5
patient 6
patient 7
patient 8
patient 9
patient 10
patient 11
alpha
other Treg
iaTreg
syn Treg
blood

Figure S3

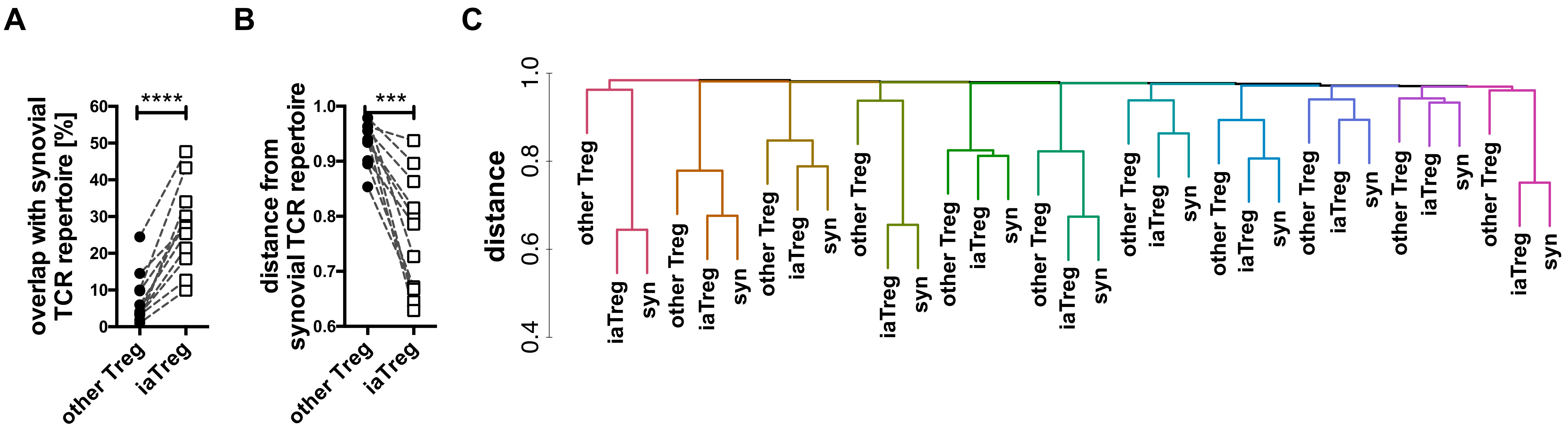

A
overlap with synovial TCR repertoire [%]
****
0
10
20
30
40
50
60
other Treg
iaTreg
B
distance from synovial TCR repertoire
***
0.6
0.7
0.8
0.9
1.0
other Treg
iaTreg
C
distance
0.4
0.6
0.8
1.0
other Treg
iaTreg
syn

Figure S4

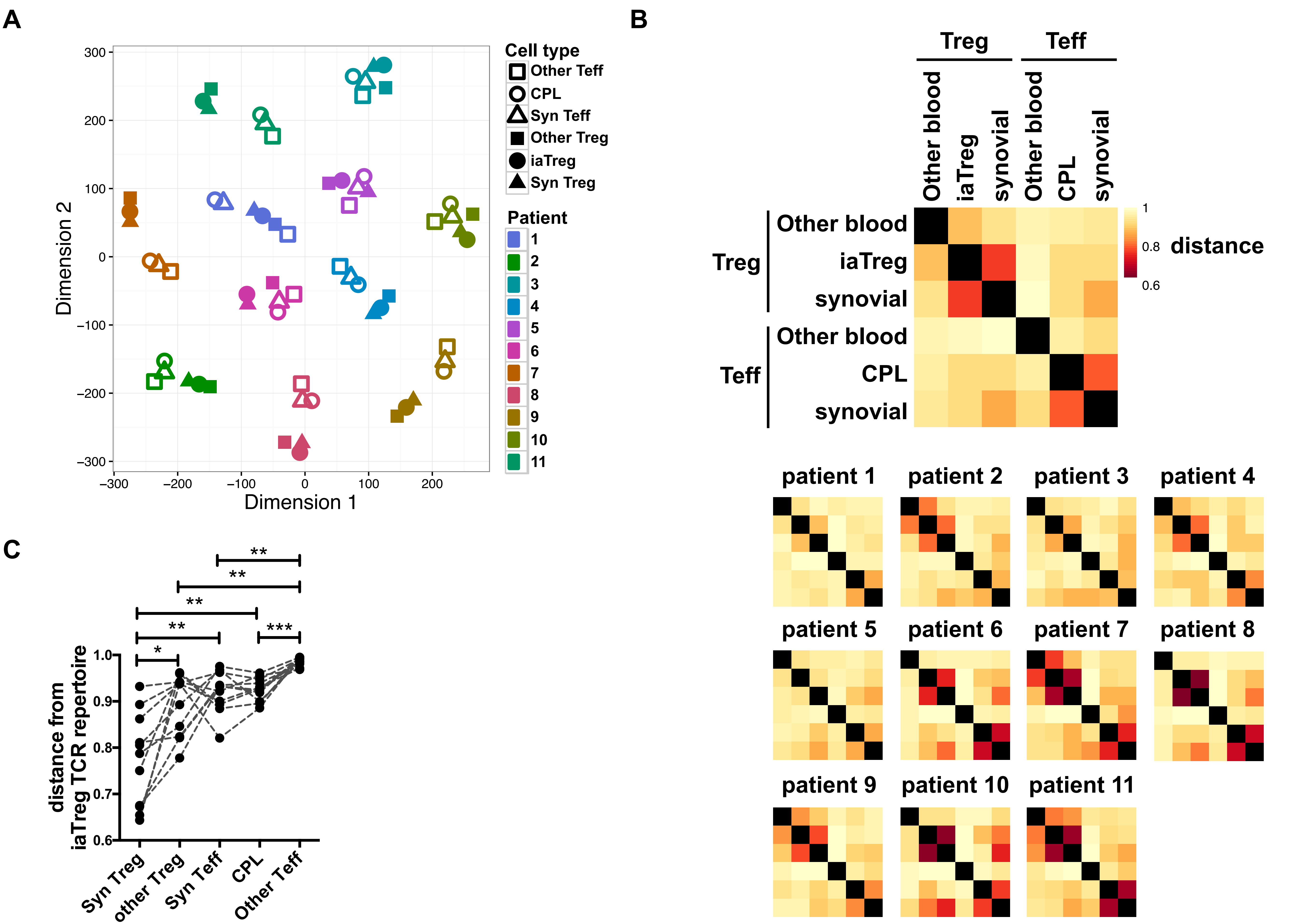
A
Cell type
Other Teff
CPL
Syn Teff
Other Treg
iaTreg
Syn Treg
Patient
1
2
3
4
5
6
7
8
9
10
11
Dimension 1
Dimension 2
B
Treg
Teff
Other blood
iaTreg
synovial
Other blood
CPL
synovial
distance
1
0.8
0.6
patient 1
patient 2
patient 3
patient 4
patient 5
patient 6
patient 7
patient 8
patient 9
patient 10
patient 11
C
distance from iaTreg TCR repertoire
Syn Treg
other Treg
Syn Teff
CPL
Other Teff
